# Safety and preliminary efficacy of sequential multiple ascending doses of solnatide to treat pulmonary permeability edema in patients with moderate to severe ARDS in a randomized, placebo-controlled, double-blind trial: preliminary evaluation of safety and feasibility in light of the COVID-19 pandemic

**DOI:** 10.1186/s13063-022-06182-3

**Published:** 2022-04-04

**Authors:** Benedikt Schmid, Peter Kranke, Rudolf Lucas, Patrick Meybohm, Bernhard Zwissler, Sandra Frank

**Affiliations:** 1 Department of Anesthesiology, Critical Care, Emergency and Pain Medicine, Wuerzburg University Hospital, Wuerzburg, Germany; 2 Vascular Biology Center, Department of Pharmacology and Toxicology and Division of Pulmonary and Critical Care Medicine, Medical College of Georgia at Augusta University, Augusta, GA 30912 USA; 3 Department of Anesthesiology, Ludwig Maximilian University Hospital, Munich, Germany; 4 Comprehensive Pulmonary Center Munich (CPC-M), Member of the German Center for Lung Research (DZL), Munich, Germany

## Abstract

**Background:**

In May 2018, the first patient was enrolled in the phase-IIb clinical trial “Safety and Preliminary Efficacy of Sequential Multiple Ascending Doses of Solnatide to Treat Pulmonary Permeability Edema in Patients with Moderate to Severe ARDS.” With the onset of the COVID-19 pandemic in early 2020, the continuation and successful execution of this clinical study was in danger. Therefore, before the Data Safety Monitoring Board (DSMB) allowed proceeding with the study and enrollment of further COVID-19 ARDS patients into it, additional assessment on possible study bias was considered mandatory.

**Methods:**

We conducted an ad hoc interim analysis of 16 patients (5 COVID-19- ARDS patients and 11 with ARDS from different causes) from the phase-IIB clinical trial. We assessed possible differences in clinical characteristics of the ARDS patients and the impact of the pandemic on study execution.

**Results:**

COVID-19 patients seemed to be less sick at baseline, which also showed in higher survival rates over the 28-day observation period. Trial specific outcomes regarding pulmonary edema and ventilation parameters did not differ between the groups, nor did more general indicators of (pulmonary) sepsis like oxygenation ratio and required noradrenaline doses.

**Conclusion:**

The DSMB and the investigators did not find any evidence that patients suffering from ARDS due to SARS-CoV-2 may be at higher (or generally altered) risk when included in the trial, nor were there indications that those patients might influence the integrity of the study data altogether. For this reason, a continuation of the phase IIB clinical study activities can be justified. Researchers continuing clinical trials during the pandemic should always be aware that the exceptional circumstances may alter study results and therefore adaptations of the study design might be necessary.

Dear Editor,

In May of 2018, the first patient was enrolled in a phase-IIB clinical trial (EUDRACT 2017-003855-47, Sponsor: APEPTICO Forschung und Entwicklung GmbH, Vienna, Austria) to assess the safety and efficacy of a novel inhalative peptide (INN: solnatide, laboratory code TIP peptide/AP301) for the treatment of moderate to severe acute respiratory distress syndrome (ARDS) [1]. A common pathophysiology of various etiologies of ARDS is pulmonary permeability edema [2]. Due to the peptide’s mechanism of action as an activator of alveolar fluid clearance and capillary barrier function in animals [3–5], and good tolerability in humans [6, 7], the current trial aims at enrolling patients with different underlying conditions leading to ARDS. When SARS-CoV-2 turned out to cause a global pandemic in the beginning of 2020, it became obvious that some of the most critically affected patients would also suffer from ARDS caused by the novel coronavirus. From the very beginning of the pandemic, there has been intensive discussion about the continuation of clinical trials in the face of the pandemic [8]. Regulatory authorities from different regions in the world have issued guidance statements regarding the conduct of clinical trials during this coronavirus 2019 (COVID-19) crisis and consensus statements have been published in some medical fields to guide the conduct of clinical trials in the light of COVID-19 [9]. In many institutions, clinical trials have been totally discontinued. In view of the potential direct significance of the intervention studied in the treatment of COVID-19 affected patients, it was initially decided by the independent Data Safety Monitoring Board (DSMB) to continue the study. However, an additional assessment by the trials’ DSMB was considered mandatory. After five COVID-19 patients had completed the trial protocol, the DSMB decided to unblind those patients’ data and to review initial clinical data in order to make sure that further enrolment of COVID-19-associated ARDS patients in the solnatide phase-IIB clinical trial would neither put the patients in undue danger nor compromise the quality of the data, e.g., due to the fact that the entity of the new disease could potentially significantly differ from the other included patients and results are not generalizable. Here, we present key findings of those five COVID-19 patients with moderate to severe ARDS irrespective of their assignment to any one treatment (solnatide or placebo) and compare them to those of eleven more patients from the same study with ARDS by causes other than SARS-CoV-2. Our aim was to identify potential differences in their clinical development over the course of the study, thus estimating the safety and feasibility of continuing the trial with SARS-CoV-2 positive patients. The data presented here originate from an ongoing clinical trial and were extracted from the eCRFpro. As this interim evaluation was not conducted according to the original protocol—but was rather based on an approved protocol amendment taking into consideration the SARS-CoV-2 pandemic—the groups of patients with and without COVID-19 were not matched in any respect.

## Baseline characteristics

Five patients were diagnosed with COVID-19 (4 male, 1 female,age 52 [49–58, median and range] years; 11 had ARDS from different causes (6 males, 5 females,age 61 [32–85] years).

At the time of screening, the acute physiology and chronic health evaluation II (APACHE II) scores differed significantly between groups (COVID: 15 [8.5–21 median and interquartile range], non-COVID: 21 [19–27], *p* = 0.022, two-sample Kolmogorov-Smirnov test, *ɑ* = 5%), suggesting that the enrolled COVID-19 patients were less critically ill than the non-COVID-19 patients. The simplified acute physiology score 3 (SAPS3) showed similar differences, yet not significantly so (COVID: 48 [46–63.5],non-COVID: 55 [49–73], *p* = 0.35). The sequential organ failure assessment (SOFA) score showed no significant differences between groups, neither at screening nor over the course of the 7-day intervention period (*F* = 0.029, *p* = 0.87, two-way ANOVA).

As the patients that were enrolled into the study were all already intubated and sedated before screening, informed consent was obtained from the patient’s personal legally designated representatives or the deferred consent procedure was used. Every local research ethics committee had given their approval to this approach before the start of the study. During a pandemic, getting written informed consent from patient’s relatives was much more complicated [10]. Due to quarantine restrictions and COVID-19 visitor bans in the hospitals, legal representatives could not be physically present to sign paper work. Therefore, informed consent had to be obtained remotely (by phone, e-mail, fax). Other alterations or protocol deviations regarding the informed consent process did not occur.

## Trial-specific outcomes

**Fig. 1:**
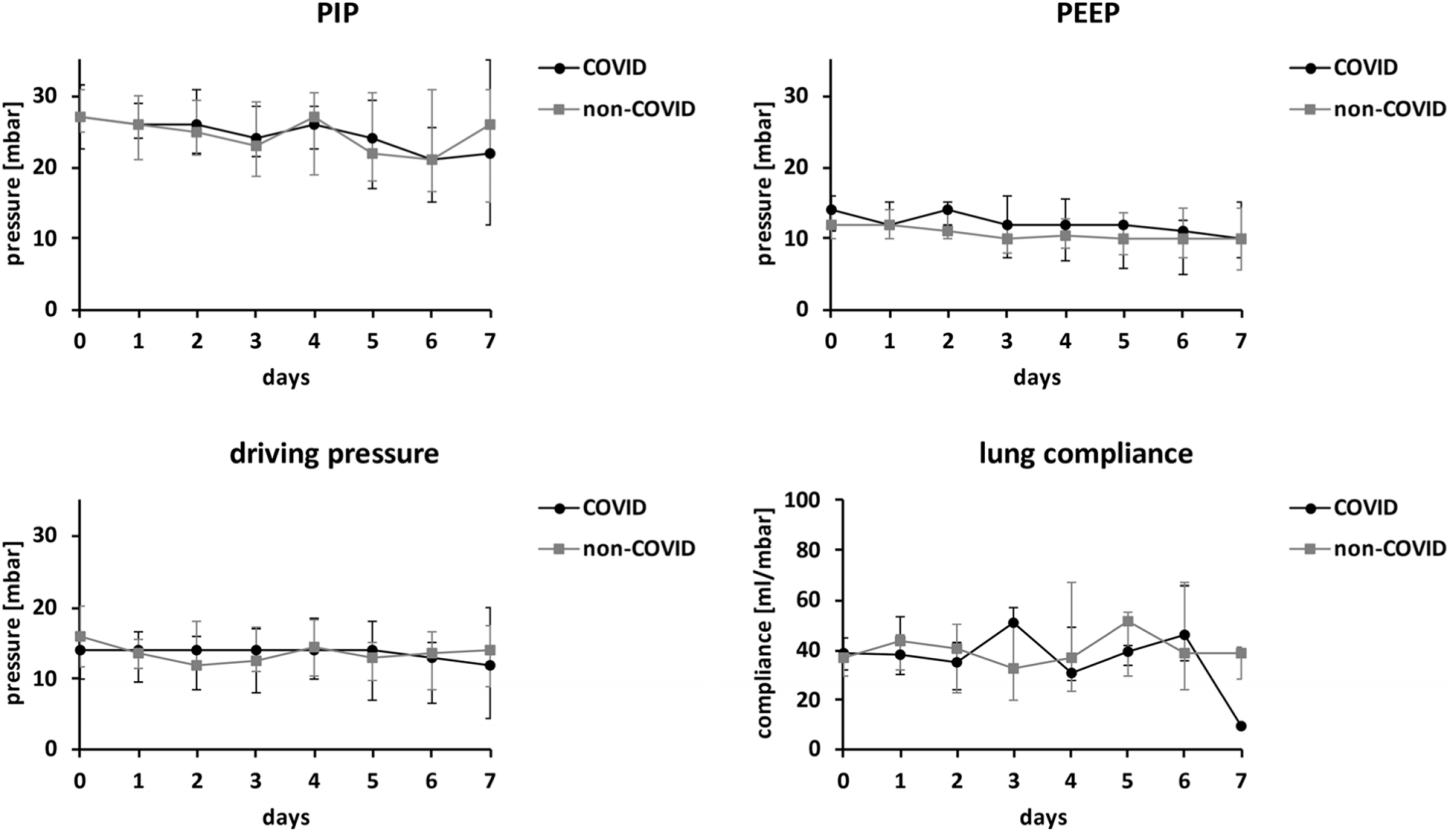
Ventilation parameters. Dots and bars denote median and IQR

Extravascular lung water index (EVLWI) and pulmonary vascular permeability index (PVPI) were measured at baseline and twice daily during intervention for seven consecutive days. There were no differences between the two groups of patients over time (EVLWI: *F* = 2.80, *p* = 0.18, PVPI: *F* = 1.75, *p* = 0.21, mixed-effects analysis). Ventilation parameters (peak inspiratory pressure [PIP], positive end-expiratory pressure [PEEP], driving pressure, and compliance) were assessed once daily during the intervention period (Fig. 1) and did not differ between groups (PIP: *F* = 0.23, *p* = 0.64, PEEP: *F* = 0.53, *p* = 0.48, driving pressure: *F* = 0.52, *p* = 0.48, compliance: *F* = 0.18, *p* = 0.69, mixed-effects analysis). Oxygenation ratio (p_a_O_2_/F_i_O_2_) was assessed once per day, and cumulative required doses of noradrenaline per 24 h were recorded. Both outcomes did not differ over the 7-day time span (oxygenation ratio: *F* = 0.03, *p* = 0.87, mixed-effects analysis,noradrenaline: *F* = 0.12, *p* = 0.73, two-way ANOVA).

## Other outcomes

Two patients died (both non-COVID), and ten made it to intensive care unit discharge (four COVID, six non-COVID) during the 28-day observation period. The distribution of ventilator-free days was widely scattered, yet not significantly different between groups (COVID: 12 [4.5–20], non-COVID: 11 [0–18], median [IQR]). Renal replacement therapy was required in one COVID and two non-COVID patients. One COVID patient and five non-COVID patients required further escalation of the ventilator support (e.g., extracorporeal membrane oxygenation).

No major deviations from study design and procedures occurred due to the COVID-19 pandemic. Special hygienic precautions had to be used during study interventions to limit the risk of infection for the health care staff. To avoid using additional protective equipment for study interventions, thorough planning, bundling, and timing of study specific assessments and procedures (e.g., ECG, EVLW measurements) was crucial. Thankfully, our study teams did not experience any critical shortage of supply chain for personal protective equipment (PPE) arising from COVID-19. Nevertheless, a major concern was running out of PPE, putting intensive care staff and patients at risk of infection [11]. Therefore, we implemented the WHO recommendations for the rational use of PPE in health care settings and temporary strategies during acute supply shortages in our patient care and study execution [12].

## Discussion

During the COVID-19 pandemic, elective surgeries, especially procedures likely to require critical care support, were postponed, in order to increase capacity for patients infected with COVID-19 [13]. As an example, an observational cohort study conducted in England and Wales showed that the total surgical activity was reduced by 33.6% in 2020, resulting in more than 1.5 million canceled operations [14]. For patients awaiting cancer surgeries, postponing or canceling needed surgical procedures during a full lockdown might lead to long-term reductions in survival [15]. Provision of elective surgery was delayed, possibly leading to increased healthcare costs [16]. As less postoperative patients were admitted to our ICUs, patient figures for secondary ARDS patients dropped.

A main cause of direct ARDS during wintertime in the northern hemisphere is influenza pneumonia. In 2020 and 2021, the emergence of the COVID-19 pandemic had an important impact on influenza virus activity. Major reductions in influenza activity were observed globally and possible reasons include non-pharmaceutical interventions (NPIs), reduced population mixing and reduced travel, but may also include virus–virus interactions, sometimes referred to as “viral interference” [17].

After WHO declared COVID-19 a public health emergency of international concern and characterized it as a pandemic on 11 March 2020, 30 countries began imposing measures to limit the spread of the disease. The timing of these measures directly correlated with a steep drop in influenza detections in 2020. Especially, encouragement of frequent hand-washing and basic hygiene measures and the advice on mask use for the general public had been effective in reducing influenza transmission [18]. Another consideration and possible hypothesis for the reduced influenza circulation is the potential interaction between the respiratory viruses SARS-CoV-2 and influenza in the same host. Circulation of multiple pathogens in a patient at the same time can result in competitive interactions, involving nonspecific, broad-acting immunity—including innate immune responses—in the host, due to prior infection or competition for the same cell types and other factors [19, 20].

Taken together, emergence of COVID-19 led to less non-COVID-19 ARDS patient admissions in the ICUs than anticipated at study initiation. Therefore, patient recruitment into the study declined, especially after the Drug Safety Monitoring Board limited the quantity of SARS-CoV-2 ARDS patients allowed to be included in the “middle dose group” of the clinical study.

In early 2020, the medical community was in urgent need for new therapeutic approaches to treat patients with the new COVID-19 disease. Researchers worldwide were under enormous time pressure in order to provide health care professionals with new knowledge as fast as possible.

Concurrently, concerns have been raised in both the scientific and lay press with regard to the quality and integrity of data, as well as with methodology and transparency of some of this research [21]. Twice as many manuscripts were submitted in the first half of 2020 than in the pre-pandemic year, with nearly the entire increase being related to COVID-19 [22]. But the pandemic’s “need for speed” increases the risk of honest error as well as misconduct, and at the end of July 2020, already more than 30 papers had to been retracted or withdrawn.

In direct comparison of COVID-19-related publications to non-COVID-publications, the COVID-19-related papers were less likely to be based on randomized controlled trials (RCTs) and more likely to use case series or other observational designs. Moreover, COVID-19 papers had smaller sample sizes, shorter follow-ups, and a higher risk of bias. Furthermore, they were more likely to have a retraction or major post-publication correction [23]. As such, these observations clearly document that the COVID-19 pandemic has profoundly changed both clinical care and research and highlight the crucial role of thoroughly planned and conducted RCTs especially in the context of a global pandemic like COVID-19.

## Summary

We conducted an ad hoc interim analysis of 16 patients from a phase-IIB clinical trial on the safety and preliminary efficacy of a novel inhalation agent for the treatment of moderate to severe ARDS, due to the unexpected challenges that arose with the onset of the global COVID-19 pandemic. We were provided with the data of five COVID-19 patients and eleven non-COVID-19 patients from the same solnatide dosing regime and similar treatment period, extracted from the eCRF. We found the COVID-19 patients to be a little less sick at baseline, which also showed in survivals over the 28-day observation period. Trial-specific outcomes regarding pulmonary edema and ventilation parameters did not differ between the groups, nor did more general indicators of (pulmonary) sepsis like oxygenation ratio and required noradrenaline doses.

In conclusion, the DSMB and the investigators did not find any evidence to support the hypothesis that patients suffering from ARDS due to SARS-CoV-2 may be at higher (or generally altered) risk when included in the trial, nor were there indications that those patients might influence the integrity of the study data altogether. For this reason, a continuation of the phase IIB clinical study activities, especially against the background of the second and third wave of the pandemic, was approved by the DSMB and is well justified from a medical point of view. We recommend that researchers take into account any specific influence of a pandemic on ongoing clinical trials and hence perform adaptations in study design to successfully complete the study and avoid bias and false interpretation of the study results.

In addition, clinical use of solnatide has been approved by the national medicines agencies in Austria and Italy within the scope of compassionate use programs for the treatment of moderate to severe COVID-19 patients.

## Data Availability

The data that support the findings of this study are available from APEPTICO Forschung und Entwicklung GmbH, Vienna, Austria, but restrictions apply to the availability of these data, which were used under license for the current study, and so are not publicly available.

## Abbreviations

APACHE II: Acute physiology and chronic health evaluation II
ARDS: Acute respiratory distress syndrome
COVID-19: Coronavirus disease 2019
EVLWI: Extravascular lung water index
IQR: Interquartile range
PEEP: Positive end-expiratory pressure
PIP: Peak inspiratory pressure
PVPI: Pulmonary vascular permeability index
RCT: Randomized controlled trial
SAPS 3: Simplified acute physiology score 3
SARS-CoV-2: Severe acute respiratory syndrome coronavirus type 2
SOFA: Sequential organ failure assessment
